# The Analytic Bilinear Discrimination of Single-Trial EEG Signals in Rapid Image Triage

**DOI:** 10.1371/journal.pone.0100097

**Published:** 2014-06-16

**Authors:** Ke Yu, Hasan AI-Nashash, Nitish Thakor, Xiaoping Li

**Affiliations:** 1 Singapore Institute for Neurotechnology, National University of Singapore, Singapore; 2 Department of Electrical Engineering, American University of Sharjah, Sharjah, United Arab Emirates; 3 Department of Biomedical Engineering, Johns Hopkins University, Baltimore, Maryland, United States of America; 4 Department of Mechanical Engineering, National University of Singapore, Singapore; Cuban Neuroscience Center, Cuba

## Abstract

The linear discriminant analysis (LDA) method is a classical and commonly utilized technique for dimensionality reduction and classification in brain-computer interface (BCI) systems. Being a first-order discriminator, LDA is usually preceded by the feature extraction of electroencephalogram (EEG) signals, as multi-density EEG data are of second order. In this study, an analytic bilinear classification method which inherits and extends LDA is proposed. This method considers 2-dimentional EEG signals as the feature input and performs classification using the optimized complex-valued bilinear projections. Without being transformed into frequency domain, the complex-valued bilinear projections essentially spatially and temporally modulate the phases and magnitudes of slow event-related potentials (ERPs) elicited by distinct brain states in the sense that they become more separable. The results show that the proposed method has demonstrated its discriminating capability in the development of a rapid image triage (RIT) system, which is a challenging variant of BCIs due to the fast presentation speed and consequently overlapping of ERPs.

## Introduction

A rapid development of brain-computer interface (BCI) related techniques has been seen in the past years. BCI system utilizing electroencephalogram (EEG) provides a shortcut of communication channel between the human brain and an external device, without conventional human’s physical response. Therefore, BCIs could be the goodwill for physically disabled patients as the promising neuroprosthetics solutions [1]–[3]. In addition, due to the advances in computation and communication, BCIs enable the new concept of gaming [4] and augment people’s performance in some applications, one of which is prioritizing images from an image pool. Fast search of target images (objects) in large-volume imagery, e.g. aerial imagery, has come to a bottleneck. That is, limit number of skillful image analysts cannot handle the increasing volume of imagery in the conventional way. Recently, the rapid image triage (RIT) technique which leverages human vision, split-second judgement capability and machine learning for EEG signal processing, has proven to be a promising solution by researchers [5]–[9]. It can be applied in various applications such as satellite image analysis and image retrieval task.

In one type of RIT, a large-scale imagery is chopped into a number of images of smaller sizes. These images are then presented to an image analyst in a sequential order at a fast speed, which is called rapid serial visual presentation (RSVP) paradigm [5], [9], [10]. During the RIT, amongst the images, some contain objects that are perceived as target objects, and hence are required to be identified for further detailed analysis [11]. In contrast, other images being irrelevant to the searching task are considered as nontargets to be disregarded. The occurrence of targets is so rare and infrequent that the searching process will induce the oddball effect [12], [13]. That is, the unique event-related potentials (ERPs) measurable on the scalp will be elicited by a recognized target image. Among these unique target ERPs, the P300 which is a prominent positive voltage deflection peaking around 300 ms after the onset of the target is the major component differing from nontarget ERPs. Hence, the backbone of RIT system is to detect, identify and dissociate these two types of ERPs.

Since ERPs are usually overwhelmed by noise such as background EEG, it is necessary to resort to various signal processing methods to improve the signal-to-noise ratio (SNR). Some of the methods are spatial decomposition based, such as principal component analysis (PCA) [14], independent component analysis (ICA) [15] and common spatial pattern analysis (CSP) [16]. Compared to PCA and ICA which extract uncorrelated/independent components, CSP is naturally more suitable for the binary classification task, as it extremizes the ratio of temporal variance of one condition over the other condition. It has been successfully applied in abundant BCI applications, including the motor imagery [17], [18], vowel speech imagery [19] and RIT [20]. In order to compensate the deficiency due to the temporal invariance, a number of CSP variants have been introduced. There are CSP variants that make use of spectral filters in conjunction with spatial filters, such as common spatio-spectral pattern (CSSP) [21], the common sparse spectral spatial pattern (CSSSP) [22] and spectrally weighted common spatial patterns (SPEC-CSP) [23]. There are also methods looking for spatio-temporal projections instead of pure spatial filters [8], [9], [24]. Being different from spectral filtering and temporal filtering, the recently proposed CSP variant, namely analytic common spatial patterns (ACSP), emphasizes on the modulation of the phases of EEG signals [25], [26]. It is accomplished by performing the spatial filtering in the complex-valued space, where the phase information is still preserved.

The phase of EEG signals is of rich unexplored implications. For instance, it has been suggested that the trial-by-trial variability of performance could be partially attributed to the fluctuation of the phase of ongoing oscillations, and the measurement of EEG phase might be useful for the prediction of perceptual and attentional variability [27]. The pre-stimulus EEG phase was claimed to affect the magnitude of the following auditory ERPs [28]. Moreover, phase is also an important property in steady-state visual evoked potentials (SSVEPs) [29]. In the context of RIT, the linkage between ERPs and phase is not apparent. However, the modulation of phase can change the morphology of ERPs in terms of magnitude and latency. And the RSVP paradigm resembles the setting of SSVEP as images are shown at specific frequency. However, it is noteworthy that spatial phase modulation proposed by ACSP, which has shown superior in classification problems such as oscillatory EEG [25] and SSVEP [26], may not be optimal in the scenario of RIT. In RIT, the target ERPs are slow potentials and can be overlapping with a number of nontarget ERPs due to the presentation speed. Therefore, a spatio-temporal phase modulation can be more useful in RIT.

In this paper, a method namely analytic bilinear discriminant analysis (ABDA) is proposed to address the spatio-temporal modulation and classification in RIT. The proposed ABDA method belongs to the linear discriminant analysis (LDA) variants, which is a traditional dimension reduction and classification method for low-dimensional feature vector. It finds the projection that maximizes the ratio of between-scatter matrix over within-scatter matrix. However, LDA becomes insufficient for handling 2D images as well as high-density EEG signals [30]. Therefore, 2D-LDA was proposed to be adapted for 2D matrix, which derives a set of orthonormal projections [31]. On the other hand, bilinear discriminant analysis (BDA) [32] and 2-dimensional linear discriminant analysis (2DLDA) [33] extend LDA by iteratively optimizing bilinear projections instead of one LDA projection. There are also trilinear (or even higher dimensional) methods, such as parallel factor analysis (PARAFAC) [34] and general tensor discriminant analysis (GTDA) [35]. These methods usually require the transformation of EEG signals into the frequency domain. Moreover, recent advances attempt to address problems like the limited sample size and doubtful distribution assumption. For instance, a variant of LDA namely enhanced Bayesian LDA (EBLDA), enlarges the sample size by incorporating unlabeled data with high probability into labeled data to refine the classification [36]. In addition, Z-LDA adaptively adjusts decision boundary to accommodate the heteroscedastic signal distribution through z-score [37]. Compared to other methods, the uniqueness of the proposed ABDA method mainly resides in: 1) exploiting the complex-valued bilinear projections, i.e. spatial and temporal projections in a complex-valued space-time domain; 2) The phases of slow ERPs are both spatially and temporally modulated, which is useful in the context of discriminating ERPs that are overlapping; 3) the spatio-temporal phase modulation demonstrated by ABDA accounts for the spatio-temporal propagation of slow ERPs. The method evaluation is conducted in the context of RIT experiments by comparing it with several competitive methods.

## Methods

### 2.1 Analytic Presentation

For a real-valued signal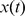, the corresponding analytic signal 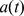 is presented as
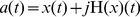
(1)where the imaginary part, 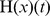, is the Hilbert transform of 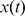. The Hilbert transform of 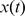 can be given by
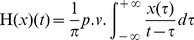
(2)where 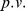 stands for the Cauchy principal value. The effect of Hilbert transform is to shift the phases of both negative frequency components and positive frequency components of a signal, but in different directions, i.e. 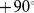 and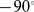, respectively. In addition, 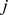 introduces another phase shift of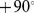 to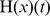. The ultimate effect is that, the negative frequency components of the analytic signal 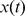 are shifted above 0 Hz. In other words, 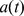 contains only positive frequency components. It is worth noting that the phases of positive frequency components of 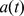 are the equivalent to the counterparts of 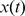.

### 2.2 Objective Function

Given the band-pass filtered EEG epochs 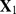 and 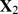 (channel
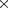
time) under two conditions (“1” for target condition and “2” for nontarget condition), the corresponding analytic presentations are denoted as 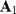 and 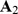, respectively. There are two bilinear projections, i.e. complex-valued spatial projection 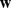 and temporal projection 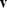. The within-class scatter 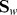 and between-class scatter 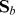 of analytic EEG epochs after temporal projecting using 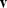 can be given as
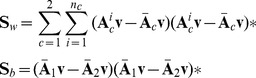
(3)where 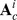 and 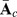 stands for the 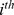 epoch and the mean matrix under condition 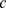, respectively. And 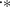 represents conjugate transpose operator.

Similarly to LDA, the objective of ABDA can be written as
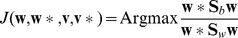
(4)


It has been shown that there is no analytical solution to a real-valued biquadratic equation [8], [32], which is a similar case for a complex-valued biquadratic equation like (4). However, there exists a sub-optimal solution, using the iterative learning.

Suppose that 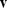 is already given, e.g. 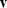 is initialized to be an identity matrix in this work. Then 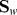 and 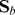 become also known and (4) can be solved by calculating the derivation by the complex-valued 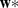. Specifically, (4) can be rewritten as
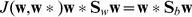
(5)In order to maximize 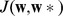, 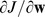 and 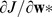 shall be set to zero. Since 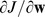 and 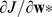 are complex conjugate transpose of each other, only one of them, e.g. 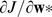, needs to be calculated:
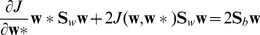
(6)Let 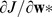 be zero, (6) can be further simplified to



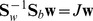
(7)(7) is exactly the same to the eigenvalue problem in LDA, the solution to which will be
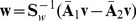
(8)


On the other hand, the within-class scatter 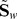 and between-class scatter 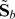 of analytic EEG epochs after spatial projecting using 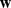 can be expressed as



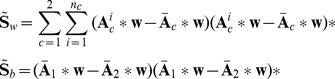
(9)And the corresponding objective function is
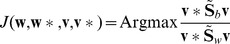
(10)


By inserting (9) into (10) and inserting (3) into (4), it can be shown that (10) and (4) are actually equivalent. Therefore, after 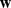 is obtained according to (8), (10) can be used to derive 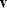 similarly by letting 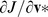 be zero, which is



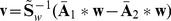
(11)By iteratively using (8) and (11), the optimal complex-valued bilinear projections 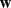 and 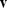 can be obtained when the ratio of between-class scatter to within-class scatter, i.e. 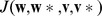, converges.

It is noteworthy that, as 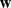, 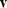 and 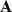 are all complex-valued, the projected data, i.e. 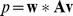, is also a complex value, which however cannot be directly used to get decision boundary. Here, 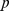 is decomposed to a 2-element vector 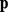, the elements of which are the real part and imaginary part of 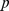. The classical LDA method is used to find a projection 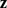 that separates 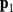 from 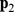.

### 2.3 Classifier

Unlike the LDA which handles the 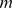-dimension to one-dimension discrimination issue [38], the proposed ABDA tackles the 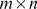-dimensional classification problem. The classifier makes use of bilinear projections, LDA projection and the bias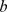, as indicated in
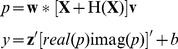
(12)where 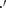 denotes the transpose operator. Moreover, 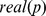 and 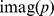 represent the real part and imaginary part of 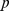, respectively.

Generally, the bias 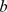 shall be chosen such that the posteriors in the projected dimension of two conditions will be equal [38]. Appropriate selection of 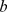 could be important if the sample size of one condition is significantly different from that of the other, which is the unbalanced classification problem.

## Experimental Setup

RIT experiments were conducted under the approval by the National University of Singapore Institutional Review Board (NUS-IRB). After providing written consent forms for participating in the experiment, 22 healthy participants, all right-handed, with normal or corrected-to-normal sight, completed the RIT experiments.

### 3.1 Experiment Design

**Figure 1:**
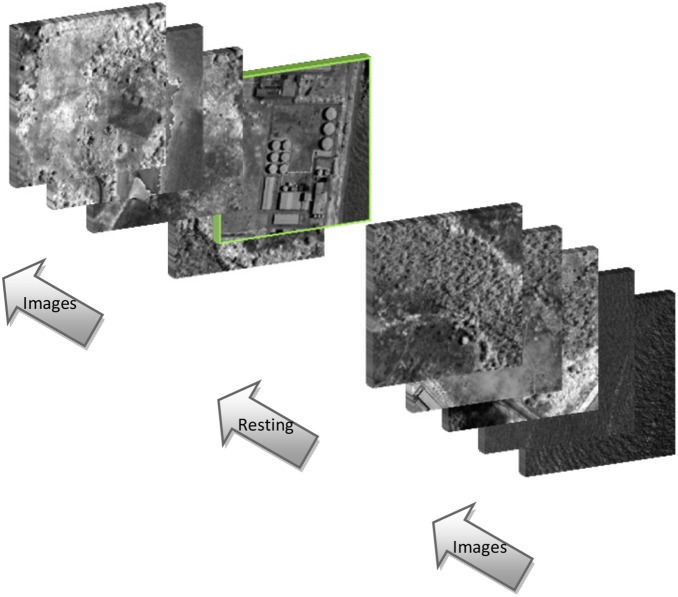
The experimental paradigm. Consecutive bursts each of which contained 50 images were serially presented and separated by a resting period. Some of the bursts contained target images.

The experiment included one training session and one testing session. In each session, aerial images were sequentially presented to a participant, following the standard RSVP paradigm [5], [9], [10] (see Figure 1). Each image lasted for 150 ms on the centre of the screen and then was replaced by the next one. There was a temporary break between every 50 images. The duration of the break was self-controlled by the participant but was caped to 10 seconds. These aerial images were of 400×400 pixels. A small amount of them (approximately 72) containing objects of interest were defined as targets, while others (over 4400) were considered as nontargets. The participant was informed that he/she should neglect nontargets but was obliged to immediately respond to the appearing targets by pressing a button.

### 3.2 Acquisition and Preprocessing

For every participant, 62-channel EEG signals were collected at 250 Hz, using an ANT amplifier (ANT B.B., Enschede, Netherlands). EEG signals were referenced to linked ears and grounded to the forehead. The 4^th^ order Butterworth filter was adopted, with the pass-band from 1 Hz to 25 Hz. The filtered signals were segmented into epochs, the time window of which starts from the onset of each image to 500 ms after the onset.

In RIT experiments, sometimes there could be a few bad channels (malfunctioning channels) and these bad channels might deteriorate the performance. Hence, ahead of training the classifier using data collected in training session, bad channels were automatically identified, which would be excluded from both training data and testing data. This was accomplished by monitoring each channel across all epochs in training session. For instance, if the absolute difference between the maximum value and the minimum value (or the mean value) is significantly large for a particular channel over 30% of total epochs, this channel would be labeled as the bad channel.

It is worth noting that the removal of identified bad channels was based on training data. However, there could be more bad channels in testing session. Thus, an additional measure was introduced. That is, every epoch was examined whether there were any suspected abnormal channels. These suspected channels would be replaced by spherical spline interpolation using neighboring functioning channels [39].

## Evaluation

With complex-valued bilinear projections, ABDA is assumed to be able to modulate the phases and the magnitudes of signals in the manner that target ERPs and nontarget ERPs become more differentiable in the context of RIT. This assumption was verified by comparing the proposed ABDA with CSP, ACSP and BDA which omits the phase modulation. All methods were applied to the EEG data collected from 22 participants in RIT experiments on a single-trial basis. The classifiers were derived using target epochs and nontarget epochs of training data, and the single-trial classification results for comparison were obtained from testing data. It is noteworthy that 4 features were extracted by CSP/ACSP using the most discriminative filters that had been derived and these features were fed to the conventional FLD classifier. The performance measure adopted was the balanced accuracy (BA) [9], which accommodates the unbalanced sample sizes between targets and nontargets. BA is defined as.
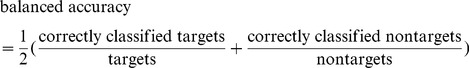
(13)


## Results and Discussion

The classification results are shown in Table 1. It can be seen that ABDA outperformed other methods for 18 out of 22 participants. The average BA achieved by ABDA was close to 90% and was 5.9% higher than CSP, 3.9% higher than ACSP and 2.5% higher than BDA, respectively. The better performance of ABDA over BDA was statistically significant in paired *t*-test, with *p*-value<0.05, which however is not significant (*p*-value = 0.017) in the *t*-test with Bonferroni correction which is conservative. Moreover, ABDA significantly surpassed CSP and ACSP, respectively, with *p*-value<0.001 in both paired *t*-test and *t*-test with Bonferroni correction. On the other hand, though BDA significantly outperformed CSP (*p*-value<0.01), its advantage over ACSP was insignificant (*p*-value>0.15) in *t*-test with Bonferroni correction.

**Table 1:** Balanced accuracies in percentage. SD stands for standard deviation. Higher BA is highlighted in bold.

	P1	P2	P3	P4	P5	P6	P7	P8	P9	P10	P11		
**CSP**	88.2	91.7	90.0	77.4	84.7	**88.1**	74.8	74.1	74.0	92.4	90.4		
**ACSP**	88.8	92.0	89.7	83.8	**92.1**	79.2	76.4	78.9	78.1	93.3	91.0		
**BDA**	89.7	90.0	81.6	84.2	90.5	86.5	**83.4**	84.2	81.7	92.2	88.5		
**ABDA**	**92.4**	**92.1**	**93.0**	**86.8**	90.7	83.3	71.3	**85.4**	**89.7**	**93.9**	**93.5**		
	**P12**	**P13**	**P14**	**P15**	**P16**	**P17**	**P18**	**P19**	**P20**	**P21**	**P22**	**Mean**	**SD**
**CSP**	82.1	84.6	91.7	79.7	86.5	81.4	86.7	85.6	82.7	74.8	82.0	83.8	±6
**ACSP**	85.1	85.2	92.3	81.0	88.6	83.3	90.2	85.5	87.8	77.7	86.6	85.8	±5
**BDA**	**88.7**	89.3	91.5	75.9	83.5	86.9	89.9	95.6	87.0	88.6	88.8	87.2	±4
**ABDA**	86.6	**91.2**	**93.8**	**89.7**	**89.3**	**91.4**	**92.2**	**95.8**	**89.3**	**90.8**	**91.4**	**89.7**	±5

**Figure 2:**
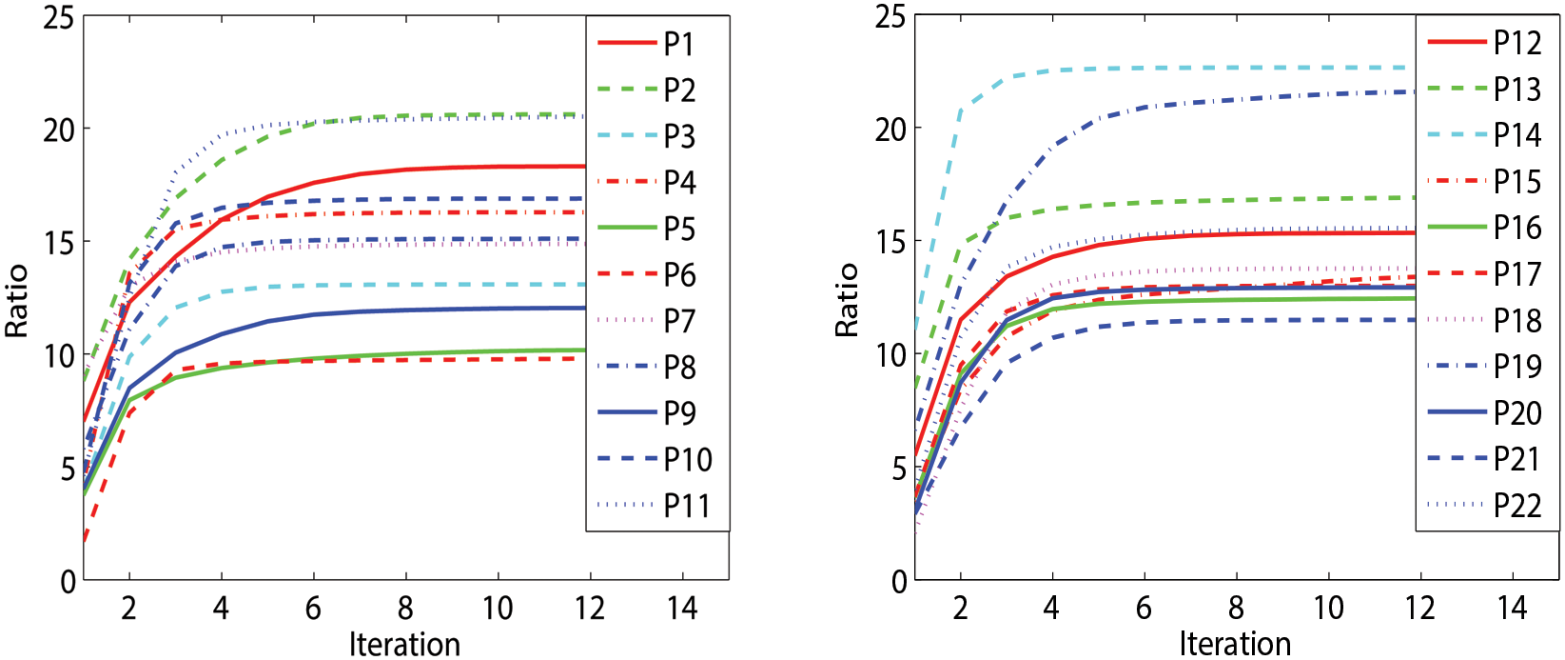
The ratios of between-class scatter to within-class scatter with respect to the iteration steps for 22 participants.

According to (4) and (10), mi and 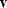 are the desirable projections that maximize the objective function. Although the derivations of 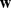 and 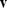 were complex-valued calculation, the ratios 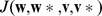 obtained during every iteration were real values (see Figure 2), as 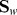 and 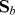 (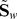 and 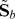) were semi-definite matrices. In Figure 2, it can be seen that for all the 22 participants, 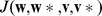 initially increased and would quickly converge to a constant value after several iteration steps. This indicates that the iterative learning was useful, and there always existed a pair of complex-valued bilinear projections which fulfilled the objective function. And most importantly, these projections could be consistently and reliably obtained for all participants.

**Figure 3:**
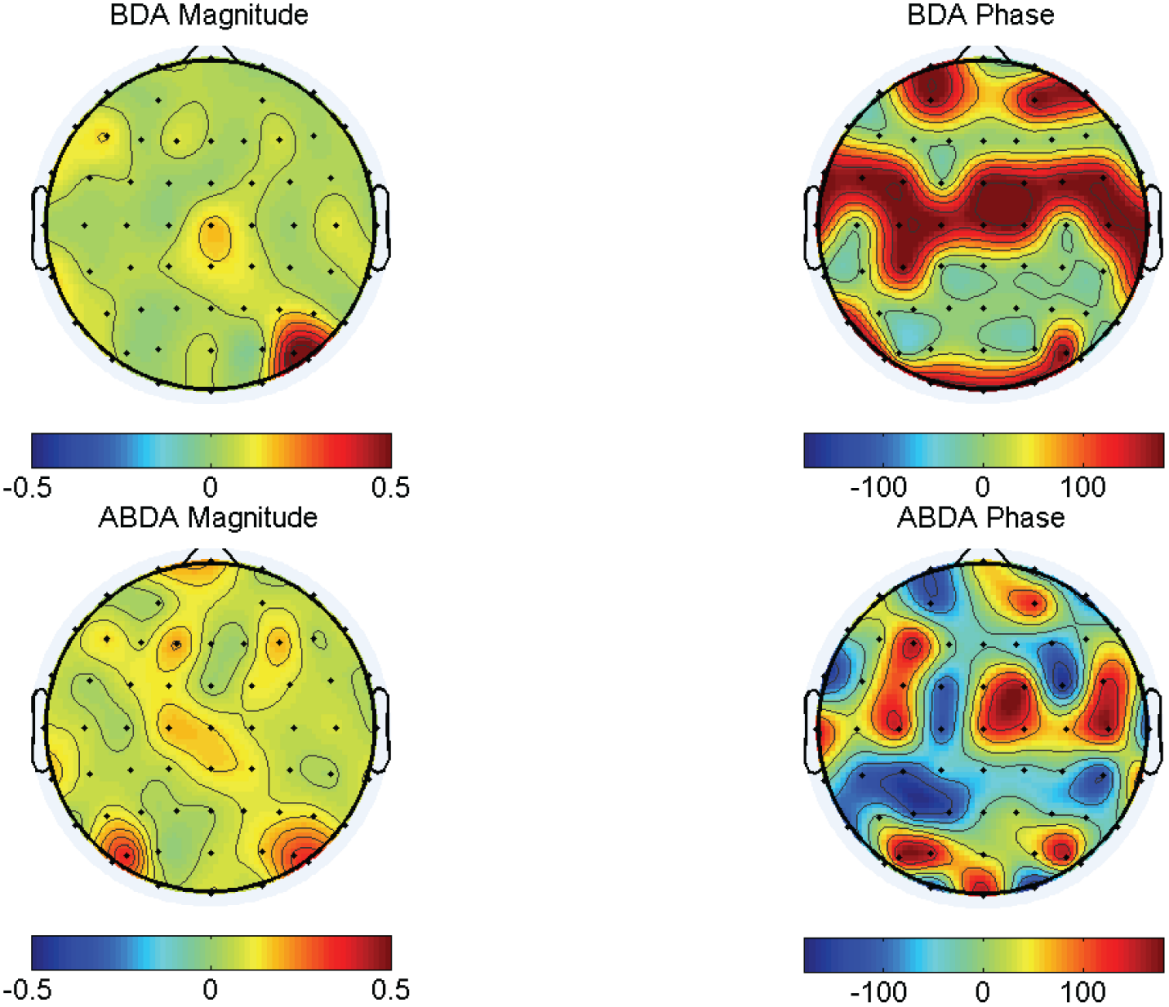
The spatial maps of Participant P22’s normalized spatial projections using BDA and ABDA methods. By Euler’s identity, a complex value could be interpreted as a combination of a magnitude component and a phase component.

The obtained ABDA spatial and temporal projections contained real parts and imaginary parts. According to Euler’s formula, a complex value has a corresponding complex exponential function consisting of two variables, i.e. magnitude and phase. In particular, the normalized spatial projection for P22 was plotted and compared to the counterpart, i.e. the BDA spatial projection, in Figure 3. It can be seen that the phases of BDA spatial projection were binary. That is, they could only be either 0° or 180°, indicating positive or negative sign, respectively. In contrast, there was more flexibility in the ABDA spatial projection. As manifested in Figure 3, the ABDA phases ranged from −180° to 180°. This freedom allowed a delicate modulation of the phases of temporal signals in each channel. The phase modulation is very useful, as it can change the morphology of signals, including amplitude and latency, to improve classification. For instance, the P300, a major signature in this RIT task, propagates from frontal to parietal areas on the scalp [40]. Such latency differences among these spatially distributed EEG channels could be estimated and utilized for denosing [20]. It can also be applied to other EEG signals such as steady-state visual evoked potentials (SSVEPs) [41]. Modulating phases of every individual channel led to the increase in the number of channels that were important for discrimination. As can be seen in Figure 3, there were much more channels of high magnitude (high weight) for ABDA in comparison to BDA. In addition, these critical channels were broadly spread such as midline, which is in accordance with the fact that P300 is measurable widely on the scalp.

**Figure 4:**
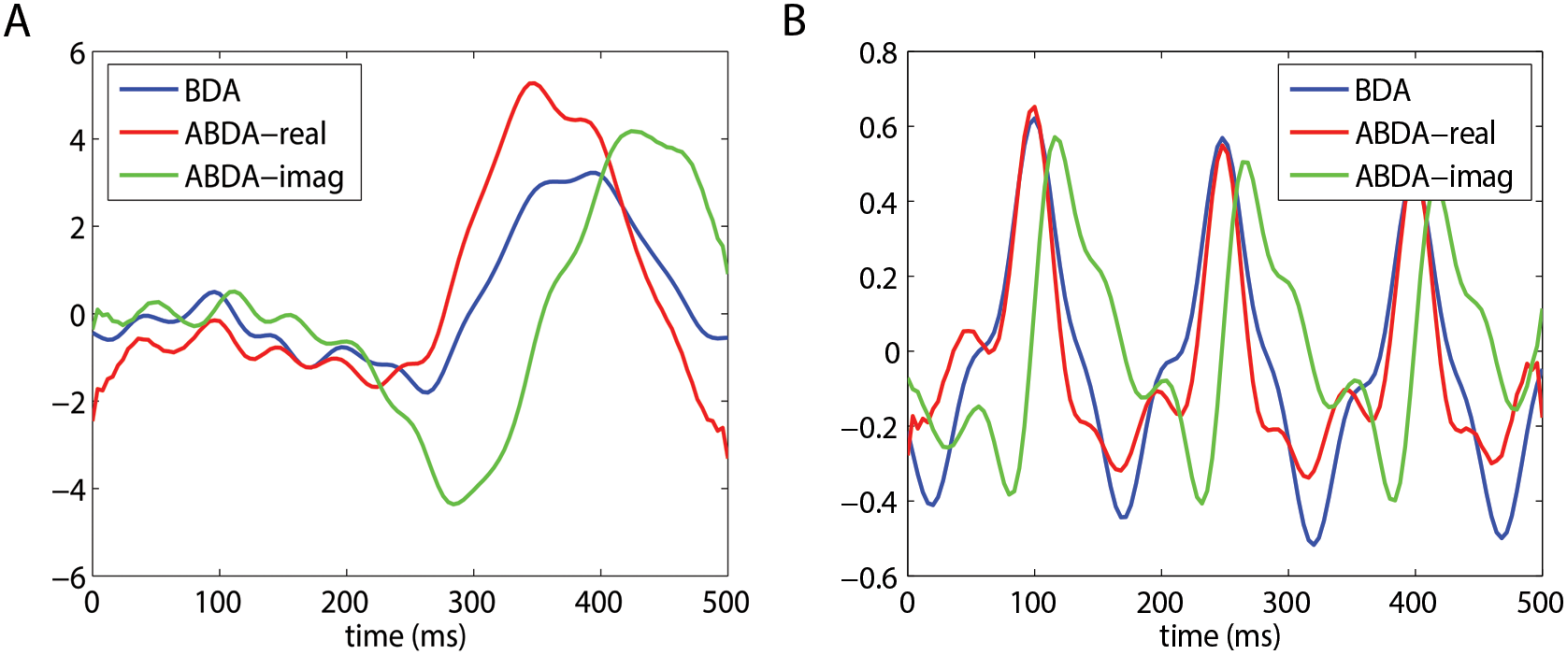
The average ERPs of Participant P22 after spatial projection using BDA and ABDA method. (a) shows the projected signals of target condition. (b) shows the projected signals of nontarget condition. ABDA-real is the real part of the projected signals, while ABDA-imag is the imaginary part of the projected signals.

The modulation offered by ABDA has a noticeable consequence which can be observed in Figure 4. After being spatial projected, both real part and imaginary part of target ERPs showed a more prominent P300 component, as compared to that in the scenario of BDA. For nontarget ERPs, the real part and imaginary part were weaker than BDA, although the difference seemed to be less significant as those in Figure 4A. Therefore, the enlarged difference between target ERPs and nontarget ERPs in Figure 4 might imply that target condition and nontarget condition became more separable by the ABDA method. However, the overall performance is determined not only by the spatial projection but also the temporal projection.

**Figure 5:**
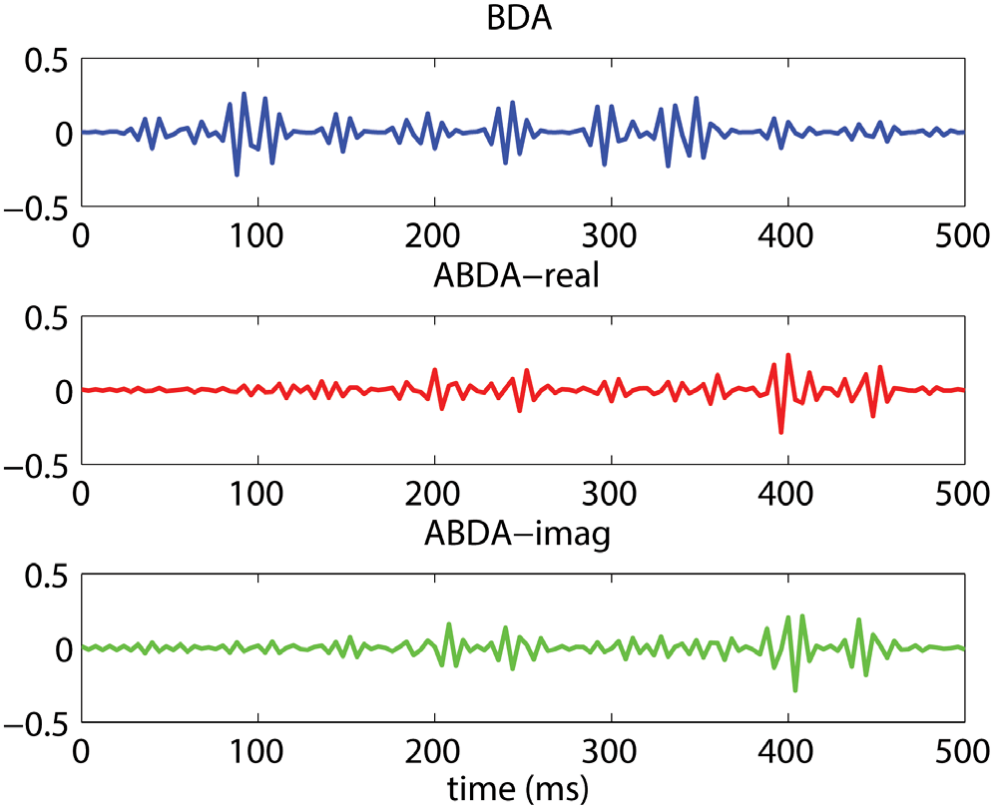
The temporal courses of Participant P22’s normalized temporal projections using BDA and ABDA methods. ABDA-real is the real part of the ABDA temporal projection, while ABDA-imag is the imaginary part of the ABDA temporal projection.

The normalized temporal projections of BDA and ABDA were plotted in Figure 5. Both BDA and ABDA temporal projections appeared to contain high frequency components. It may be due to the fact that, the temporal resolution (time point) was higher than the spatial resolution (the number of electrodes) and a regularization term was not adopted during the iteration learning. From the perspective of the waveform, BDA imposed heavier weights on the first half of the time window, i.e. between 0 ms and 350 ms. These weights could be meaningful. As can be seen in Figure 4A, the projected target ERPs (blue line) peaked at 350 ms, which matched the corresponding weights in Figure 5, indicating a linkage between BDA temporal projection and BDA spatial projection. On the other hand, the ABDA method focused mainly on the late stage of the target ERPs, i.e. 400 ms, and the waveform looked very clean before 200 ms. This is in line with the work of Gerson et al. [5], where the prominent discriminating activities were observed after 350 ms. It also followed the peak of spatially projected signals in Figure 4A, in particular the ABDA-imag (green line).

**Figure 6:**
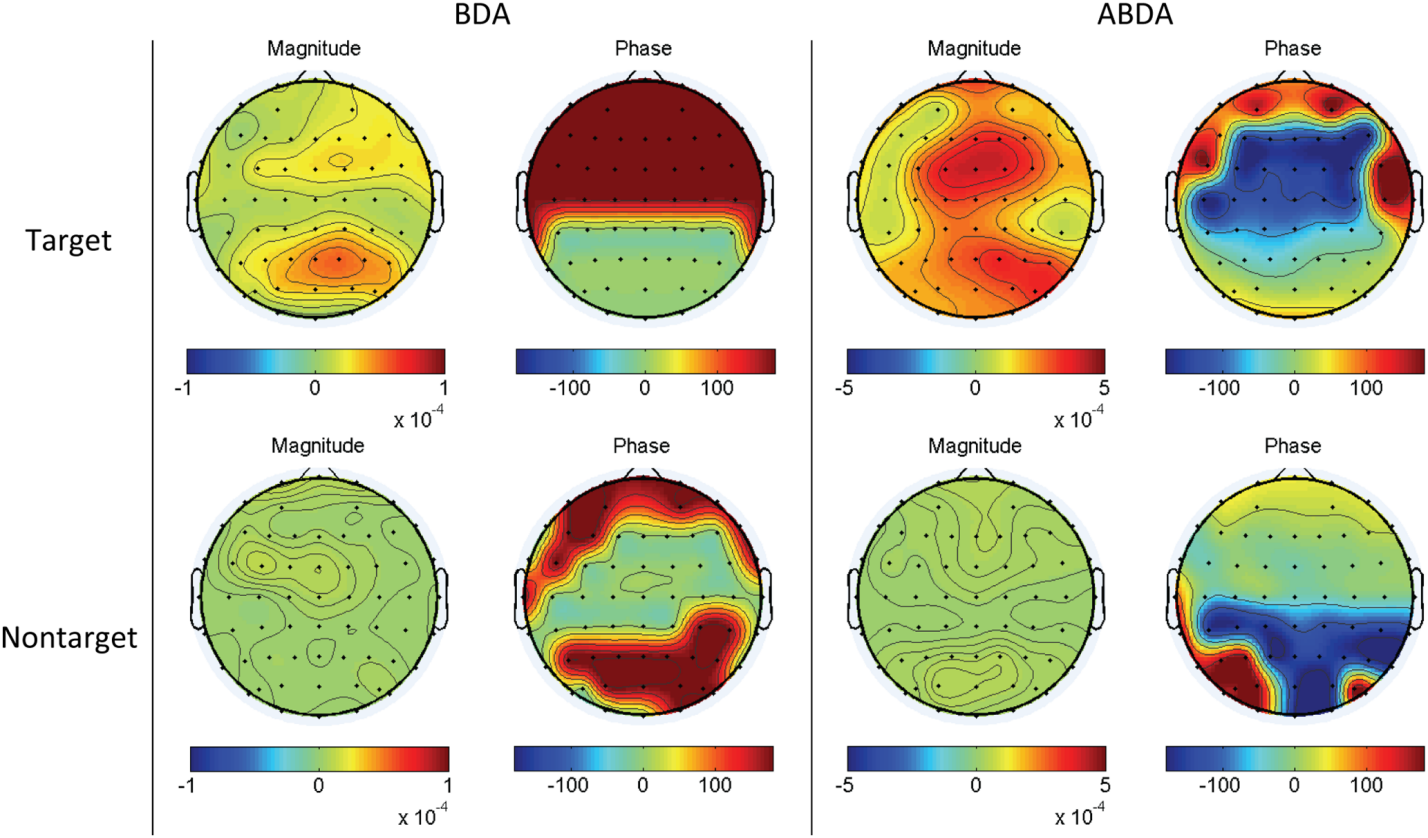
The average EEG spatial maps of Participant P22 after temporal projection using BDA and ABDA methods. By Euler’s identity, a complex value could be interpreted as a combination of a magnitude component and a phase component.

With the temporal projections as shown in Figure 5, the projected spatial topographies of two conditions were obtained in Figure 6. For both methods, the magnitude difference between target condition and nontarget condition were apparent, suggesting a strong discriminating capability of the temporal projection. With respect to the comparison between BDA and ABDA, there was a similar observation in Figure 3. That is, ABDA seemed to exploit a larger region for classification, from frontal, central to parietal and occipital, which was demonstrated by the magnitude mappings of BDA and ABDA under target condition. Furthermore, it is interesting to note that the phase mapping of ABDA under target condition partially showed the gradual propagation pattern of target ERPs, e.g. P300. It is known that the latency of P300 is shorter over frontal areas and longer over parietal areas [42]. At the first glance, it seemed that there was a noticeable phase gap between frontal areas (dark red) and central areas (dark red). However, it is noteworthy that phase is periodic and 180° is equivalent to −180°. Therefore, the phase difference between the frontal and the central areas was actually small. In general, it could be said that the color-coded phases of ABDA in Figure 6 progressively changed from dark red, dark blue to yellow color along the scalp, resembling the P300 latency changes. The phase mapping in Figure 6 indicated the phase difference between the signals in EEG channels in a quantitative manner. Unlike ABDA, BDA did not account for the ERP propagation. For instance, in Figure 6 under target condition, all phases in the frontal and central areas were 180° (negative), and the rest were 0° (positive).

**Figure 7:**
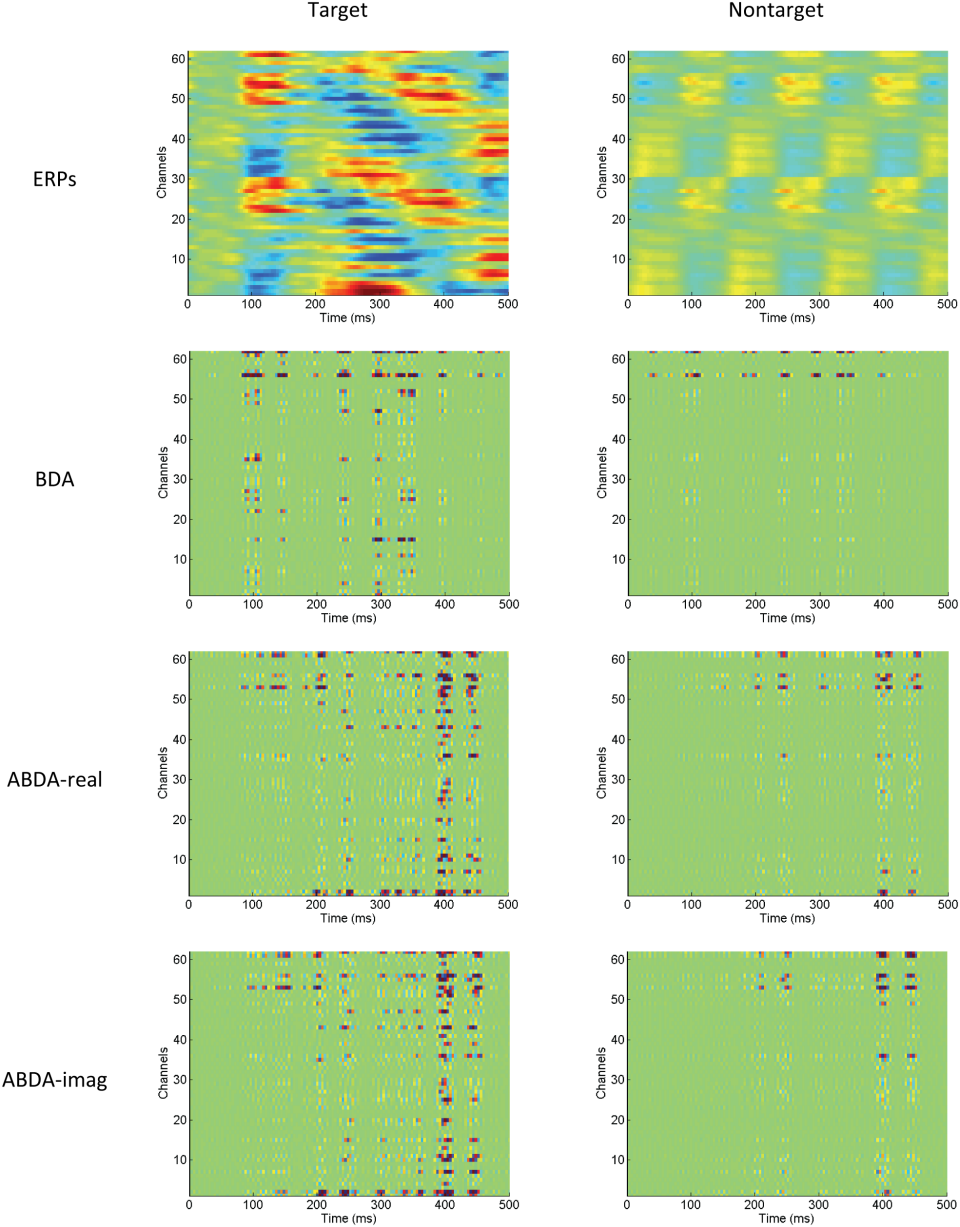
The plotting of average ERPs of Participant P22 and the outcomes after the spatio-temporal projection by the BDA and ABDA methods. The first row shows the target ERPs and nontarget ERPs along all the 62 channels, and are under the scale of [−5 5]. The other three rows are the element-wise products of ERPs (or the corresponding analytic representation) and the spatio-temporal projection. The scale is [−0.1 0.1]. ABDA-real is the real part of the ABDA element-wise products, while ABDA-imag is the imaginary part of the ABDA element-wise products.



Since there is an inherent relation between the iteratively optimized spatial projection and temporal projection, evaluating the spatial projection and temporal projection in a separate way may be insufficient for viewing the big picture. Figure 7 illustrates the combined effect of bilinear projections. Given the ERPs (see the first row in Figure 7), the element-wise product for ABDA was calculated using.
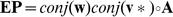
(14)where 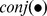 stands for the conjugate function and 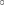 represents the element-wise product multiplication operator. The formula for BDA was simpler:

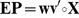
(15)It is worth noting that the summation of all the elements of 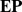 is equivalent to 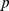 in (12). According to the 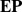 of ABDA and 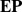 of BDA in Figure 7, it can be seen that ABDA mainly relied on the late stage of target ERPs, whilst the early ERP components were favored by BDA. Moreover, a larger number of channels and time points were ‘highlighted’ by ABDA to distinguish target condition from nontarget condition. On the other hand, BDA depended on relatively limited spatio-temporal signal segments. Additionally, there is a kind of ‘texture’ at the first row of Figure 7, which can be attributed to the propagating process of ERPs on the scalp. Such a texture is also observable in the 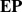 of BDA (the second row), which however, became absent in the 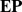 of ABDA. The absence of this texture should be the result of the phase and magnitude modulation introduced by the complex-valued bilinear projections, which counteracted the latency differences among channels.

## Conclusions

In this study, ABDA, the analytic bilinear discriminant analysis, a linear discriminant analysis originated method, is proposed and has been applied to the development of the RIT system. The results showed that, without transforming into frequency domain, the ABDA method is capable of modulating the phases and magnitudes of slow ERP signals that are overlapping with other ERPs, using the coupling of complex-valued spatial projection and complex-valued temporal projection. The complex-valued bilinear projections accommodated the spatio-temporal phase variations of ERPs, and consequently enabled a better usage of high-density EEG measurement to perform the classification task. With the ABDA, the RIT tests have showed an average accuracy increase of 2.5% over that with the BDA method and also outperformed CSP and ACSP.
